# Spondylolisthesis and mismatch deformity affect outcomes after total knee arthroplasty

**DOI:** 10.1186/s13018-023-03605-y

**Published:** 2023-03-02

**Authors:** William L. Sheppard, Daniel Chiou, Alexander Upfill-Brown, Akash Shah, Eghosa Edogun, Adam Sassoon, Don Y. Park

**Affiliations:** 1 Department of Orthopedic Surgery, University of California, Los Angeles, 1250 16th St Suite 2100, Santa Monica, CA 90404 USA; 2 David Geffen School of Medicine, University of California, Los Angeles, 10833 Le Conte Ave, Los Angeles, CA 90095 USA

**Keywords:** Degenerative spondylolisthesis, Mismatch, Total knee arthroplasty, Outcomes

## Abstract

**Background:**

Little published data currently exist regarding the potential relationships between spondylolisthesis, mismatch deformity, and clinical outcomes following total knee arthroplasty (TKA). We hypothesize that preexisting spondylolisthesis will result in decreased functional outcomes after TKA.

**Methods:**

This retrospective cohort comparison of 933 TKAs was performed between January 2017 and 2020. TKAs were excluded if they were not performed for primary osteoarthritis (OA) or if preoperative lumbar radiographs were unavailable/inadequate to measure the degree of spondylolisthesis. Ninety-five TKAs were subsequently available for inclusion and divided into two groups: those with spondylolisthesis and those without. Within the spondylolisthesis cohort, pelvic incidence (PI) and lumbar lordosis (LL) were calculated on lateral radiographs to determine the difference (PI–LL). Radiographs with PI–LL > 10° were then categorized as having mismatch deformity (MD). The following clinical outcomes were compared between the groups: need for manipulation under anesthesia (MUA), total postoperative arc of motion (AOM) both pre-MUA or post-MUA/revision, incidence of flexion contracture, and a need for later revision.

**Results:**

Forty-nine TKAs met the spondylolisthesis criteria, while 44 did not have spondylolisthesis. There were no significant differences in gender, body mass index, preoperative knee range of motion (ROM), preoperative AOM, or opiate use between the groups. TKAs with spondylolisthesis and concomitant MD were more likely to have MUA (*p* = 0.016), ROM < 0–120 (*p* < 0.014), and a decreased AOM (*p* < 0.02) without interventions.

**Conclusion:**

Preexisting spondylolisthesis by itself may not have adverse effect clinical results following TKA. However, spondylolisthesis increases the likelihood of developing MD. In those with both spondylolisthesis and concomitant mismatch deformities, patients had statistically and clinically significantly decreased in postoperative ROM/AOM and increased need for MUA. Surgeons should consider clinical/radiographic assessments of patients with chronic back pain who present for total joint arthroplasty.

**Level of evidence:**

Level 3.

## Background

Both low back pain and osteoarthritis (OA) are the leading causes of functional impairment and years lived with disability for adults above the age of sixty [1–11]. However, these issues are not mutually exclusive. Degenerative changes and limitations in range of motion in the knee are correlated with changes in lower lumbar spinal alignment and pain [12, 13]. Patients with advanced knee OA who are considering total knee arthroplasty (TKA) often have concomitant symptoms of lower back pain or radiating pain to the lower extremities [10, 11, 14–18]. Among patients, changes in vertebral disk heights at the lower lumbar levels correlate with knee pain [19]. Degenerative spondylolisthesis, or anterior translation of a vertebral segment, also commonly occurs at the lower lumbar region [20–22]. Thus, it is important to understand, identify, and analyze the overlap in symptoms of low back pain and knee arthritis for better outcomes and patient satisfaction.

Severe knee OA is known to cause compensatory reductions in lumbar lordosis and flexion contractures that lead to more spinal misalignment and symptoms [13, 23–27]. In patients who present with concomitant lower back and knee pain, the order of treatment is usually determined by the severity and location of symptoms, activities of daily living, and preferences [28]. Yet, TKA patients with coexisting and lumbar spine symptoms had worse preoperative functional scores compared to those without lumbar spine symptoms [14]. Despite literature citing that some patients received resolution of their lumbar symptoms after their total hip arthroplasties (THA), similar findings are still debated with regard to TKA [29]. It can be argued that these concomitant symptoms may confound perioperative TKA functional outcomes and patient satisfaction.

Studies have shown that spinal sagittal mismatch deformity and lumbar stenosis negatively affect TKA, but there is little evidence regarding spondylolisthesis affecting TKA outcomes [30, 31]. Thus, we performed a retrospective review to analyze the relationship of predisposing spondylolisthesis and TKA outcomes among patients receiving TKAs at our institution. We hypothesize that patients with preexisting spondylolisthesis will have worse outcomes and postoperative function than those with normal lumbar alignment preoperatively.

## Methods

### Study design and spinal alignment measurements

An analysis of 933 TKAs in 845 patients was performed at a single health care system from January 2017–2020 for primary OA. Patients were excluded for a lack of spinal imaging (714), having inadequate preoperative plain-film radiographs to perform spondylolisthesis measurements (108), and prior lumbar spine intervention (17). Ninety-three TKAs performed in 81 patients by seven different arthroplasty surgeons met inclusion criteria. All spinopelvic parameters were obtained in accordance to institutional protocol [28]. There were no statistical differences in materials used or postoperative radiographic parameters in accordance with prior studies from our group [28, 29].

A clinical follow-up through at least three months post-operation was sustained in all but 3 patients. The patients who did not reach the 3-month follow-up period achieved full functionality of the knee (> 120-degree arc of motion). TKAs were then separated into two groups. One group included patients with spondylolisthesis and the other included patients without spondylolisthesis.

### Demographics and outcomes

Patients’ age, sex, and body mass index (BMI) were recorded for the study. Range of motion (ROM) < 0–120, arc of motion (AOM), and preoperative opiate use were potential confounders accessed for in group comparison and regression analyses. Radiographic and material analyses of knee implants were conducted by two authors.

**Fig. 1:**
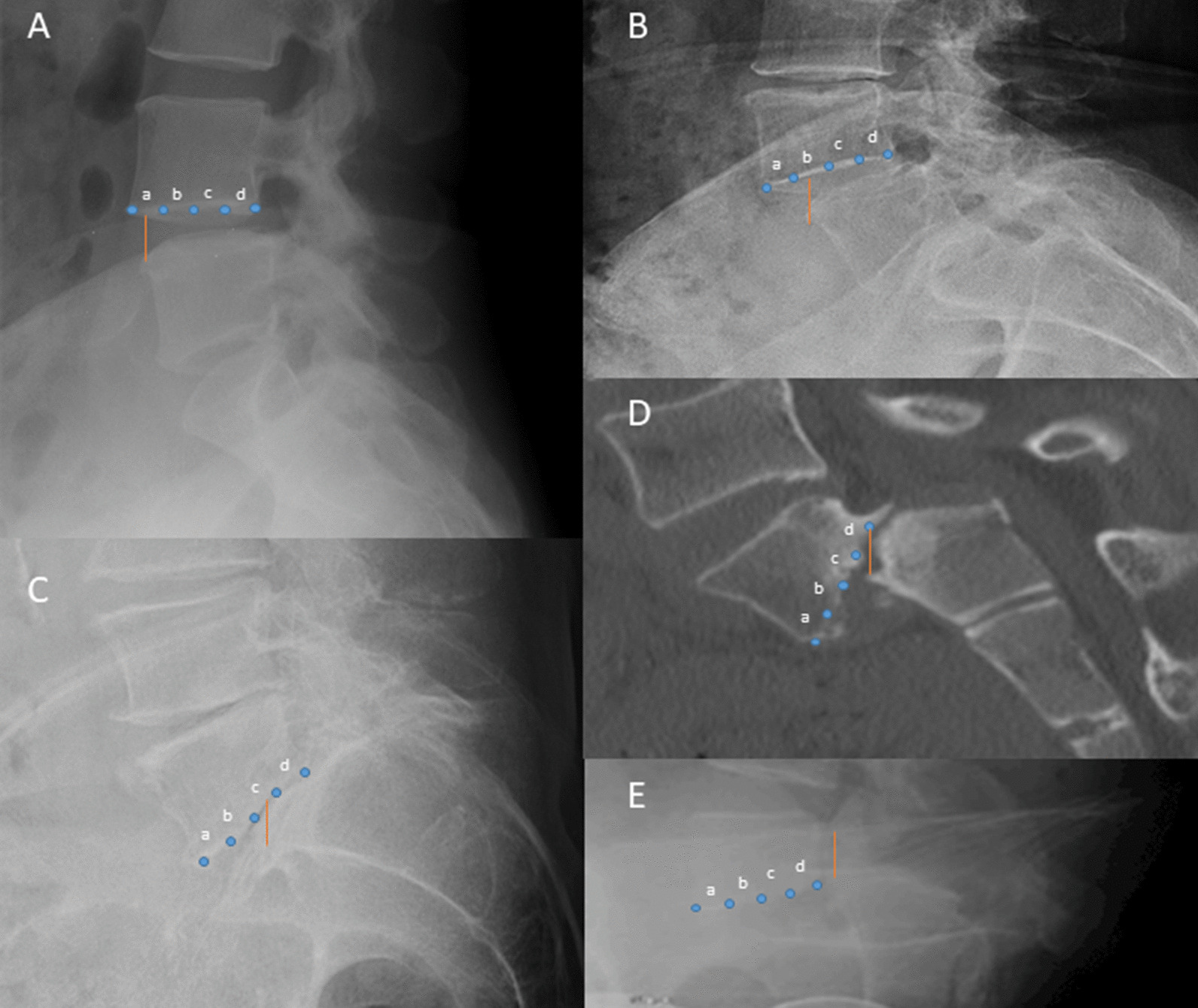
Meyerding classification explained [30]. **A** Represents grade 1 spondylolisthesis with anterior translation of L4 on L5 within the “a” range of < 25%. **B** Represents grade 2 spondylolisthesis with anterior translation at the same level within the “b” range of 25–50%. **C** Represents grade 3 spondylolisthesis with anterior translation of L5 on S1 within the “c” range of 50–75%. **D**, **E** were not involved in this study but represent 75–100% anterior displacement within the “d” range, and spondyloptosis (>100% anterior displacement), respectively. These measurements are with respect to the anterior aspect of the inferior vertebral body. Similarly, the posterior aspect of the superior vertebral body may be used to assess grade as the percentages of listhesis remain consistent

The presence of flexion contracture, postoperative AOM (difference between maximum extension and flexion), postoperative ROM < 0–120, manipulation under anesthesia (MUA) utilization, the difference between the preoperative and postoperative arc of motion (ΔAOM) were the primary outcomes measured. Data on range of motion were obtained during pre- and postoperative office visits by the attending surgeons, orthopedic residents, and physician assistants. On the lateral lumbar radiographs, the degree of spondylolisthesis was measured and graded based on the Meyerding grading scale (Fig. 1A–E): grade 1, < 25% displacement, grade 2, 25–50% displacement, grade 3, 50–75% displacement [30]. Grade 4 or 5 was not noted in this study group. In addition, pelvic incidence (PI) and lumbar lordosis (LL) were calculated on lateral radiographs to determine the difference (PI–LL) [28]. Radiographs with PI–LL > 10° were then categorized as having mismatch deformity (MD), as normal is < 10° by definition [24–28, 30].

### Statistical analysis

Comparative analyses of two independent groups were completed with chi-squared and t tests, with the utilization of a two-tailed method for categorical and continuous variables, respectively. Correlation coefficients were used to assess the association between spinal alignment measures. The functional relationship of sagittal alignment parameters and outcomes of interest was measured by regression analysis while controlling for other confounders. Significance was determined as *p* < 0.05. Analysis was carried out in R version 3.3.1. (R Core Team. R Foundation for Statistical Computing. Vienna, Austria).

## Results

Of the 93 TKAs included in our analysis, there were 44 patients identified with spondylolisthesis (S) and 49 patients without spondylolisthesis (NS) (Table 1). Approximately 40% of the subjects with spondylolisthesis presented with a Meyerding grade 1 slip, 17% with a Meyerding grade 2 slip, and one patient with a Meyerding grade 3 slip. A total of 4 patients had both a grade 1 and grade 2 slip at 2 different levels. The majority of slips occurred at the L4-L5 and the L5-S1 levels. Between the cohorts, there were no significant differences noted with respect to sex, age, laterality, or BMI. However, there was a significant difference between instances of osteoporosis in S patients versus NS patients. Preoperative characteristics such as ROM < 0–120, AOM, and opiate use were not statistically significant between groups. There were three revisions (two in the NS and one in the S group due to patellar osteophytes), which occurred 1 year postoperatively. There was 1 complication: 1 retained drain fragment in the spondylolisthesis group.

**Table 1:** Spondylolisthesis versus no spondylolisthesis: patient demographics, preoperative characteristics, spinal deformity measurements, postoperative outcomes

	No spondylolisthesis	Spondylolisthesis	*p* value
	*n* = 44	*n* = 49	
*Patient characteristics*			
Sex, female	79.5% (35)	77.3% (34)	0.64
Age, mean	69.0	71.4	0.19
BMI, mean	29.7	28.6	0.33
Osteoporosis/penia	22.7% (10)	53.1% (26)	0.002
Laterality (right)	20	25	0.60
*Pre-op characteristics*			
ROM less than 0–120	45.5% (20)	57.1% (28)	0.26
Arc of motion, mean	111	113	0.52
Opiate use	11.4% (5)	12.2% (6)	0.90
Coronal deformity	70.5% (31)	65.3% (32)	0.60
*Measurements*			
Mismatch deformity (PI–LL > 10)	12.5	22.2	< 0.001
Pelvic incidence	53.3	61.7	< 0.001
Lumbar lordosis	43.9	49.4	0.21
Pelvic tilt	16.9	24.7	< 0.001
Sacral slope	37.2	36.6	0.81
*Outcomes*			
MUA	0.0% (0)	12.2% (6)	0.016
ROM less than 0–120	68.2% (30)	42.9% (21)	< 0.001
Arc of motion, mean	118	111	0.024
Delta arc of motion, mean	110	104	0.21
Revisions	4.55% (2)	0% (0)	0.13
Flexion contracture	11.4% (5)	12.2% (6)	0.90

Six patients in the spondylolisthesis group underwent MUA for postoperative stiffness (*p* = 0.016), while no TKAs in the NS group underwent subsequent MUA. Differences in incidence of postoperative ROM not reaching < 0–120 and postoperative AOM were reduced in the S group (*p* < 0.05). There was a 16° reduction in mean AOM for the S group (range, 25–130°; standard deviation (SD) = 18.0) when compared to those for the NS (range, 110–135°; SD = 4.9).

For TKAs requiring MUA, prior to manipulation, the mean AOM was 87.8° (range, 25–120°; SD = 25). Median AOM was 115° pre-manipulation. After manipulation, the mean AOM increased to 102° (range, 188–120°; SD = 14). Median post-manipulation AOM was 103°. This 29° increase in AOM after manipulation (measured at 5 weeks, 3, 7, 10, 20, and 28 months post-manipulation) did not match the mean 123° AOM for the NS group.

Linear regression models were used to analyze AOM and ΔAOM (Table 2). Patients with increased MD have a 15.3°smaller change in AOM than patients with no MD (*p* < 0.001). There was no significant difference in AOM for patients with spondylolisthesis for any grade when controlling for mismatch deformity. Multivariate regression was used to analyze reduced terminal extension and ROM < 120° (Table 2). In the case of reduced terminal extension (stiffness), there was also no significant differences found among the different groups and demographics. Most notably, the odds of concomitant spondylolisthesis for those with MD were 2.8 when compared to those without spondylolisthesis (CI 1.2–6.5, *p* = 0.02). Furthermore, mismatch deformity was inversely higher in those with any grade of spondylolisthesis, with 9.7° of sagittal imbalance noted on average compared to the control group (CI 4.6–14.8, *p* = 0.0003).

**Table 2:** Regression analysis: spondylolisthesis and mismatch deformity affect clinical outcomes after TKA

	Arc of motion		*p*	Delta arc of motion		*p*	Terminal extension lacking		*p*
	Est	95% CI		Est	95% CI		OR	95% CI	
Spondylolisthesis	− 4.51	(− 11.8, 2.78)	0.22	− 5.21	(− 65.7, 55.3)	0.87	0.61	(0.11, 3.37)	0.56
Preoperative AOM	0.07	(− 0.17, 0.3)	0.57	− 8.52	(− 18.3, 1.25)	0.089	0.99	(0.95, 1.05)	0.83
Mismatch deformity	− 15.27	(− 22.01, − 8.53)	0.001	− 11.95	(− 21, − 2.89)	0.011	12.3	(1.24, 122)	0.030
Sacral slope	0.27	(− 0.06, 0.6)	0.11	0.32	(− 0.12, 0.77)	0.16	1.01	(0.93, 1.09)	0.90
Osteoporosis	0.66	(− 6.86, 8.18)	0.86	4.36	(− 5.73, 14.5)	0.40	0.97	(0.17, 5.6)	0.97
Opioids	− 8.36	(− 18.73, 2.01)	0.11	5.13	(− 8.12, 18.4)	0.45	0.77	(0.07, 8.49)	0.83
Body mass index (BMI)	− 0.12	(− 0.77, 0.53)	0.71	0.01	(− 0.86, 0.89)	0.97	1.03	(0.88, 1.2)	0.72
Age	0.04	(− 0.38, 0.47)	0.84	0.13	(− 0.45, 0.71)	0.66	1.04	(0.94, 1.14)	0.461
Female	− 4.66	(− 12.2, 2.88)	0.22	− 6.73	(− 16.9, 3.45)	0.19	1.98	(0.34, 11.4)	0.44

## Discussion

The purpose of this study was to determine if spondylolisthesis of the lumbar spine would affect TKA outcomes clinically and functionally. We predicted that those with spondylolisthesis would have worse clinical outcomes. We subsequently identified significant relationships between spondylolisthesis with concomitant mismatch deformity and adverse outcomes following TKA. Interestingly, 12.2% of the S group underwent MUA (*p* = 0.016), with an average AOM of 111° (*p* < 0.05). No patients in the NS group required a MUA, with the mean postoperative AOM being 118°, well above the minimum recommendation for independent living (~ 110°) [32]. Furthermore, those in the S group did not meet postoperative ROM 0–120° approximately 43% of the time (*p* = 0.014). The data remained consistent when controlling for confounders through regression analysis, which demonstrated a significant increase in the likelihood of MUA, arc of motion, and ROM for those with MD (*p* = 0.017 and *p* = 0.013, respectively). However, on regression, these findings were heavily influenced by the presence of MD. Furthermore, the odds of concomitant spondylolisthesis for those with MD were 2.8 when compared to those without spondylolisthesis (*p* = 0.02). The incidence of MD was inversely higher in those with any grade of spondylolisthesis, with 9.7° of sagittal imbalance noted on average compared to those without spondylolisthesis (*p* = 0.0003). This alone would place patients near the threshold/definition of MD.

With respect to the MD groups, several findings remained consistent: (1) Mean AOM was 16° less when MD was noted, (2) approximately 77% of those with MD failed to have a ROM of 0°-120° or better, (3) nearly 25% of those with MD developed a flexion contracture of 6.25° on average, and (4) there was a 5° reduction in AOM from pre- to post-op for those with MD. All findings were independent of spondylolisthesis presence on regression analysis.

There are no studies currently analyzing the relationship between spondylolisthesis and functional outcomes after TKA. Vigdorchik et al. showed that in their cohort of 78 patients, those with spinal deformities (defined as PI–LL ≥ 10°) had limited knee ROM after TKA and only had improved flexion but not extension after MUA [33]. Sheppard et al. also showed in their cohort of 53 patients that those with mismatch deformities (defined as PI–LL > 10°) were more likely to require MUA, develop flexion contractures postoperatively, and have a decreased AOM by 16° [32]. Similarly, this study demonstrates that patients with mismatch deformities, in conjunction with spondylolisthesis, have poorer postoperative outcomes in TKA. Patients with both spondylolisthesis and mismatch deformity may compensate with their lower extremities to maintain overall sagittal balance, as it is well described that patients with mismatch deformity retrovert their pelvis, hyperextend the hips, and flex the knees [29, 30]. The knee flexion compensation, which likely is chronic and longstanding, may contribute to the postoperative results of TKA seen in this study. It is more likely that the mismatch deformity is what predisposes patients to poorer TKA outcomes, rather than the spondylolisthesis “component.” There is emerging evidence that the deformity also affects the clinical results and complications with THA.

There are several limitations to this study. First the surgeons’ preferences accounted for thresholds for MUA and implant selection, although the AOM < 90 is the standard for manipulation at our institution. In addition, this is a single-center retrospective study. Thus, the data were obtained from chart review and did not permit repeated measurements. Only 93 TKAs met inclusion criteria, underpowering this study’s analysis and potentially contributing to selection bias in our population.

In conclusion, concomitant spondylolisthesis and sagittal mismatch deformity may negatively impact clinical outcomes after total knee arthroplasty. While preexisting spondylolisthesis alone may not have adverse effect clinical results following TKA, those with spondylolisthesis and concomitant mismatch deformities have statistically significant decreases in postoperative ROM/AOM and increased need for MUA. The presence of any grade of degenerative spondylolisthesis increases the odds of developing MD. The presence of increased severity of MD has been shown to negatively influence postoperative outcomes after TKA. Arthroplasty surgeons should be aware of this relationship between spinal malalignment and poor TKA outcomes and may consider referral to a spine surgeon prior to TKA for evaluation of lumbar spondylolisthesis, especially if the patient exhibits a coexisting mismatch deformity.

## Data Availability

The datasets generated and/or analyzed during the current study are not publicly available due to the regulations by our Institutional Review Board, but can be made available from the corresponding author by request.

## Abbreviations

TKA: Total knee arthroplasty
OA: Osteoarthritis
PI: Pelvic incidence
LL: Lumbar lordosis
MD: Mismatch deformity
MUA: Manipulation under anesthesia
AOM: Arc of motion
BMI: Body mass index
ROM: Range of motion
THA: Total hip arthroplasty
∆AOM: Difference between preoperative and postoperative AOM
S: Spondylolisthesis (group)
NS: No spondylolisthesis (group)
SD: Standard deviation
CI: Confidence interval
Est: Estimate
OR: Odds ratio
*p*: *p* Value
